# Reliability, Accuracy, and Comprehensibility of AI-Based Responses to Common Patient Questions Regarding Spinal Cord Stimulation

**DOI:** 10.3390/jcm14051453

**Published:** 2025-02-21

**Authors:** Giuliano Lo Bianco, Marco Cascella, Sean Li, Miles Day, Leonardo Kapural, Christopher L. Robinson, Emanuele Sinagra

**Affiliations:** 1 Anesthesiology and Pain Department, Foundation G. Giglio Cefalù, 90015 Palermo, Italy; giulianolobianco@gmail.com; 2 Anesthesia and Pain Medicine, Department of Medicine, Surgery and Dentistry “Scuola Medica Salernitana”, University of Salerno, 84081 Baronissi, Italy; 3 National Spine and Pain Centers, Shrewsbury, NJ 07702, USA; sli@treatingpain.com; 4 Department of Anesthesiology, Texas Tech University Health Sciences Center, Lubbock, TX 79430, USA; miles.day@ttuhsc.edu; 5 Carolinas Pain Institute, Winston-Salem, NC 27103, USA; lkapuralmd@gmail.com; 6 Anesthesiology, Perioperative, and Pain Medicine, Harvard Medical School, Brigham and Women’s Hospital, Boston, MA 02115, USA; 7 Gastroenterology and Endoscopy Unit, Fondazione Istituto San Raffaele Giglio, 90015 Cefalù, Italy; emanuelesinagra83@googlemail.com

**Keywords:** spinal cord stimulation, ChatGPT, artificial intelligence, patient education, chronic pain management, neuromodulation, healthcare communication

## Abstract

**Background:** Although spinal cord stimulation (SCS) is an effective treatment for managing chronic pain, many patients have understandable questions and concerns regarding this therapy. Artificial intelligence (AI) has shown promise in delivering patient education in healthcare. This study evaluates the reliability, accuracy, and comprehensibility of ChatGPT’s responses to common patient inquiries about SCS. **Methods:** Thirteen commonly asked questions regarding SCS were selected based on the authors’ clinical experience managing chronic pain patients and a targeted review of patient education materials and relevant medical literature. The questions were prioritized based on their frequency in patient consultations, relevance to decision-making about SCS, and the complexity of the information typically required to comprehensively address the questions. These questions spanned three domains: pre-procedural, intra-procedural, and post-procedural concerns. Responses were generated using GPT-4.0 with the prompt “*If you were a physician, how would you answer a patient asking*…”. Responses were independently assessed by 10 pain physicians and two non-healthcare professionals using a Likert scale for reliability (1–6 points), accuracy (1–3 points), and comprehensibility (1–3 points). **Results:** ChatGPT’s responses demonstrated strong reliability (5.1 ± 0.7) and comprehensibility (2.8 ± 0.2), with 92% and 98% of responses, respectively, meeting or exceeding our predefined thresholds. Accuracy was 2.7 ± 0.3, with 95% of responses rated sufficiently accurate. General queries, such as “*What is spinal cord stimulation*?” and “*What are the risks and benefits*?”, received higher scores compared to technical questions like “*What are the different types of waveforms used in SCS*?”. **Conclusions:** ChatGPT can be implemented as a supplementary tool for patient education, particularly in addressing general and procedural queries about SCS. However, the AI’s performance was less robust in addressing highly technical or nuanced questions.

## 1. Introduction

ChatGPT is a large-scale artificial intelligence (AI) model with 175 billion parameters, released in November 2022 by OpenAI [1]. Built on the natural language processing (NLP) technology known as the “generative pre-trained transformer” (GPT), this large language model (LLM) generates conversational text responses to given inputs. Its potential applications in healthcare have gained significant traction, with emerging examples in neuromodulation, pain management, mental health support, and patient education [2,3,4,5,6]. Specifically, LLM tools like ChatGPT have demonstrated promise in addressing patient queries across various medical specialties, offering innovative ways to communicate complex information and enhance patient understanding [7,8]. In anesthesia, intensive care unit (ICU), and perioperative care, AI models have been utilized to streamline patient–provider communication, provide real-time decision support, and facilitate personalized patient education [9,10]. For instance, AI-driven chatbots have assisted in preoperative assessments, postoperative care instructions, and managing patient anxiety by providing timely and accurate information [11,12,13]. These applications underscore the potential of these AI-based tools to improve clinical workflows, enhance patient satisfaction, and support healthcare professionals in delivering high-quality care.

Despite the advancements, the integration of AI in clinical settings presents several challenges, including ensuring the accuracy and reliability of AI-generated information, addressing concerns related to data privacy, and mitigating the risk of information overload for patients [14,15]. Additionally, there is an ongoing debate regarding the optimal use cases for AI in healthcare and the extent to which these tools can supplement or augment the roles of healthcare providers [16]. Identifying and addressing these challenges is crucial for the effective implementation of AI technologies in sensitive and high-stakes environments [9,10].

Spinal cord stimulation (SCS) is an advanced neuromodulation therapy widely used to manage chronic pain conditions that have not responded to more conservative therapies [17,18]. Commonly utilized for conditions such as failed back surgery syndrome, complex regional pain syndrome, and other neuropathic pain disorders, this therapy aims to provide significant pain relief and improve the quality of life [19,20]. Despite its effectiveness for some patients, SCS requires careful patient selection, as not all individuals may benefit from the procedure. Additionally, patients often have numerous concerns and questions about the treatment process, including aspects related to trialing, system implantation, potential risks, and long-term management [21,22,23]. Addressing these concerns through clear, accurate, and comprehensible information is essential for fostering informed decision-making and enhancing treatment confidence [24,25,26,27].

In this context, sophisticated AI tools like ChatGPT can serve as valuable supplementary resources for patient education. Specifically, by providing accessible, reliable, and consistent information, AI chatbots have the potential to bridge gaps in patient understanding and support healthcare providers in delivering personalized care [26,28,29,30]. However, the effectiveness of such tools in specific clinical scenarios, such as SCS, remains underexplored. Existing studies have primarily focused on the general utility of AI in patient education, with limited emphasis on specialized procedures and the nuanced information needs of patients undergoing complex treatments [31,32,33].

This study aims to investigate the feasibility of using a chatbot to address patient concerns and improve the comprehension of a complex medical procedure within the perioperative care framework.

## 2. Materials and Methods

### 2.1. Study Objectives

This study evaluated the reliability, accuracy, and comprehensibility of GPT 4.0 responses to common patient questions about SCS. A secondary objective was to assess the potential utility of ChatGPT in improving patient understanding and acceptance of SCS by addressing frequently asked questions across pre-procedural, intra-procedural, and post-procedural domains.

### 2.2. Query Strategy

A modified Delphi-based strategy was implemented to define the relevant questions. Specifically, our study employed a structured methodology that focused on (1) the selection of patient questions and (2) the assessment criteria used by evaluators.

The selection process was designed to identify clinically relevant and frequently asked questions regarding SCS from a patient perspective. This was achieved through a modified Delphi approach, integrating multiple sources including clinical expertise, a literature review, and online patient resources. The research team, consisting of interventional pain specialists with extensive experience in SCS, compiled a preliminary list of common patient concerns based on direct clinical interactions and consultations. For the literature review, a targeted review of patient education materials and published studies on SCS was conducted to identify recurring themes in patient inquiries [34]. Finally, publicly available patient education platforms, including hospital websites and medical forums, were examined to capture real-world patient concerns [35]. The final selection prioritized thirteen of the most common patient questions regarding SCS implants.

These selected questions, detailed in Table 1, were categorized into three domains: pre-procedural (questions 1–4), intra-procedural (questions 4–8), and post-procedural (questions 9–13).

Subsequently, each question was submitted to GPT 4.0 on 25 July 2024 using the prelude “*If you were a physician, how would you answer a patient asking…*”. The AI-generated responses were recorded for evaluation. Consequently, an in-depth review process was implemented to ensure coverage of clinical nuances and patient-specific scenarios in AI-generated responses.

### 2.3. Evaluation Process

The responses were assessed by a panel of 12 participants, including 10 interventional chronic pain physicians with expertise in SCS and 2 non-healthcare professionals experienced in patient education for chronic pain management.

Each evaluator conducted an independent assessment of the AI-generated responses using a structured Likert scale to ensure objective and standardized evaluation across three critical dimensions:

- *Reliability* (1–6 scale): It assessed the consistency, coherence, and trustworthiness of each response. A score of 1 indicated an unreliable, inconsistent, or potentially misleading response, while a score of 6 denoted a highly dependable response, aligned with established clinical guidelines, and demonstrated minimal risk of misinformation;
- *Accuracy* (1–3 scale): This criterion measured the degree of factual correctness concerning current medical knowledge on SCS. A response receiving a 1 contained incorrect or misleading statements; a 2 was deemed generally accurate but with minor omissions or ambiguities, and a 3 signified highly precise information that was fully aligned with current clinical evidence;
- *Comprehensibility* (1–3 scale): This category evaluated the clarity, readability, and ease of understanding from a patient perspective. A score of 1 indicated a response that was too technical or difficult for a layperson to understand, a 2 represented a moderately clear response that might require some clarification, and a 3 was awarded to responses that were well-structured, easy to follow, and free of unnecessary medical jargon.

Finally, the results were analyzed to determine trends in ChatGPT’s performance across different domains of patient inquiries. To ensure that ChatGPT’s responses met minimum quality thresholds for practical patient education, we established predefined acceptability cutoffs based on prior studies assessing similar constructs. A response was deemed acceptable if it achieved the following:

- ≥4 points in reliability (indicating strong consistency and trustworthiness);
- ≥2 points in accuracy (ensuring at least a satisfactory level of factual correctness);
- ≥3 points in comprehensibility (confirming that the response was sufficiently clear for patient comprehension).

### 2.4. Word Length Analysis

To address concerns regarding information overload, an analysis of the word length of ChatGPT’s responses was conducted. This analysis aimed to assess whether the length of responses varied significantly across different procedural domains and its potential impact on patient comprehension. The goal was to ensure that responses were neither too brief to lack substance nor too lengthy to cause information overload. The word count for each response was calculated using standard text processing tools.

### 2.5. Statistical Analysis

Quantitative data were expressed as means with standard deviations (SD). Statistical analyses were performed using the IBM SPSS v. 26 software to evaluate agreement among participant ratings and identify patterns in ChatGPT’s performance. The study was exempt from the Institutional Review Board review as no patient-related data was involved.

## 3. Results

The overall mean reliability of ChatGPT’s responses was 5.1 ± 0.7, with 92% of responses scoring ≥ 4 (Table 2). Q7 and Q12 received slightly lower reliability scores of 4.6 ± 0.8 and 4.8 ± 0.9, respectively, while Q3 and Q9 achieved the highest ratings, with mean scores of 5.6 ± 0.5 and 5.5 ± 0.5.

The overall mean accuracy was 2.7 ± 0.3, with 95% of the responses rated as sufficiently accurate (≥2). Q5 and Q11 scored the highest for accuracy at 2.9 ± 0.1, whereas Q10 and Q13 were rated lower, with scores of 2.5 ± 0.3 and 2.6 ± 0.4, respectively.

**Figure 1:**
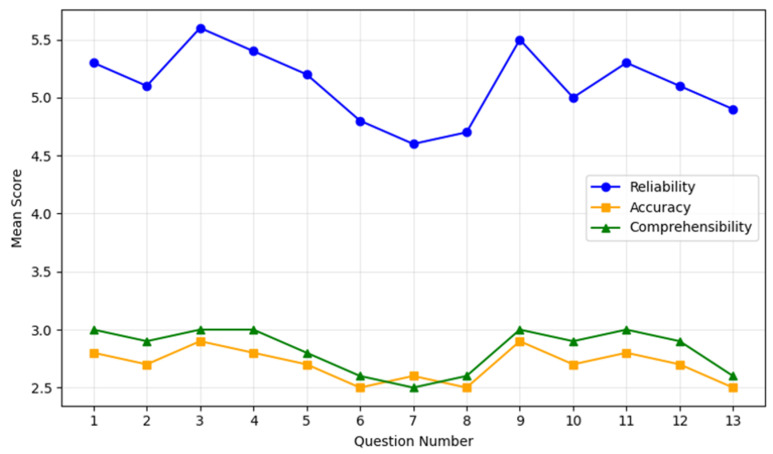
ChatGPT performances on patients’ questions. The chart illustrates the mean scores for reliability (blue line), accuracy (orange line), and comprehensibility (green line) across the 13 most common patient questions regarding spinal cord stimulation. Each point on the lines corresponds to the average score (with standard deviation bars omitted for clarity) assigned by the evaluators. The *X*-axis enumerates the questions from 1 to 13, while the *Y*-axis shows the mean score within each evaluated domain. Overall, reliability demonstrates consistently higher scores (generally above 5.0), indicating that most responses were rated highly trustworthy. Accuracy and comprehensibility remain in the mid-to-high range (around 2.5–3.0), reflecting an acceptable precision of information and a relative ease of understanding for patients. Minor dips in accuracy and comprehensibility scores appear on more technical questions (e.g., Questions 6–8), consistent with the challenges of addressing specialized or complex content.

Comprehensibility was rated 2.8 ± 0.2 on average, with 98% of responses meeting or exceeding the threshold of ≥3. Q4 and Q9 achieved the highest comprehensibility scores of 3.0 ± 0.0, while Q13 received the lowest rating of 2.5 ± 0.4, with evaluators suggesting simpler language for improved clarity (Figure 1).

These results highlight differences in performance between general and technical queries. Higher scores were observed for procedural and post-procedural questions, while responses to technical questions, such as those addressing waveform types and device troubleshooting, showed room for improvement (Table 2).

The analysis of word length revealed that the average number of words per response varied across the three procedural domains. Pre-procedural responses averaged 150 ± 20 words; intra-procedural responses averaged 180 ± 25 words, and post-procedural responses averaged 200 ± 30 words (Table 2).

## 4. Discussion

This study demonstrates ChatGPT’s capability to provide reliable and comprehensible answers to common patient questions regarding SCS. The high reliability (5.1 ± 0.7) and comprehensibility (2.8 ± 0.2) scores suggest that ChatGPT performs well in general patient education, particularly for procedural expectations and post-operative management. These findings highlight AI’s transformative potential in addressing gaps in healthcare communication, enabling the scalability and standardization of information delivery.

Similar studies in other medical fields have evaluated the effectiveness of AI models for patient education, reinforcing the versatility and potential of such technologies. For instance, in oncology, AI-driven chatbots have been utilized to provide patients with information about treatment options, side effects, and coping strategies, resulting in improved patient satisfaction and reduced anxiety levels [36,37]. In diabetes management, AI tools have facilitated personalized education on blood sugar monitoring, diet, and exercise, leading to better disease management and patient adherence to treatment plans [38]. These studies collectively underscore the broad applicability of AI in enhancing patient education across various specialties, aligning with our findings in the context of SCS. For example, ChatGPT’s responses to questions like “*What is spinal cord stimulation?*” and “*What are the risks and benefits?*” were rated highly for clarity and alignment with clinical explanations. Therefore, it emerges that ChatGPT can distill complex medical information into accessible, patient-friendly language. Such capabilities are consistent with findings from other studies where AI effectively simplifies intricate medical concepts, thereby enhancing patient understanding and engagement [5].

However, the model demonstrated limitations when addressing technical or context-specific topics, such as waveform types (Q7) and advanced troubleshooting (Q12), where scores were lower. This likely reflects ChatGPT’s reliance on publicly available general knowledge, which may not include detailed or nuanced medical content. These findings confirm results from previous studies, demonstrating that AI can perform well in general education but may fall short in handling specialized topics or adhering to rapidly evolving medical guidelines [37,38].

Moreover, the word length analysis revealed that the average number of words per response varied across the three procedural domains. Pre-procedural responses averaged 150 ± 20 words; intra-procedural responses averaged 180 ± 25 words, and post-procedural responses averaged 200 ± 30 words (Table 2). While more detailed explanations post-procedure may enhance comprehensibility, excessively long responses in technical domains can contribute to information overload, potentially overwhelming patients [39]. Balancing the depth of information with clarity is crucial to ensure that patients receive comprehensive yet manageable information.

The challenges of integrating ChatGPT into clinical practice extend beyond its ability to provide accurate responses. The model’s training data may limit its relevance in fields like SCS, where advancements are frequent. Additionally, the model cannot provide culturally or linguistically tailored responses, which are essential for addressing the diverse needs of patient populations. Ethical and legal considerations, such as the risk of misinformation and ensuring accountability, must also be addressed before AI tools like ChatGPT can be widely adopted [9,40].

Recent studies have demonstrated AI’s role in revolutionizing care for critical illnesses. For example, in the management of cardiogenic shock, AI algorithms have been employed to enhance diagnostic accuracy and optimize treatment protocols, leading to improved patient outcomes [41,42]. Furthermore, a recent state-of-the-art review on the applications of AI in pulmonary hypertension has highlighted the significant impact of AI in enhancing diagnostic precision, personalizing treatment plans, and improving patient monitoring [33]. This review underscores the adaptability of AI tools like ChatGPT in addressing the specific informational and educational needs of patients with complex medical conditions. By tailoring responses to the unique challenges associated with pulmonary hypertension, AI demonstrates its capacity to support both patients and healthcare providers in specialized clinical contexts, thereby reinforcing the relevance of our study in the broader landscape of AI-driven patient education. These advancements illustrate how AI can augment clinical decision-making and streamline workflows in high-stakes environments, highlighting the potential for similar integrations in the management of chronic pain through SCS.

### 4.1. Limitations

Several limitations of this study should be acknowledged. First, the small sample size and the focus on responses generated by a single LLM platform may limit the generalizability of the findings. While ChatGPT provides valuable insights, its capabilities cannot fully represent other AI models, such as Google Gemini (formerly Bard) or Microsoft Co-Pilot, which leverage related but distinct underlying technologies [43]. Including comparisons across multiple platforms would provide a more comprehensive evaluation and reduce the risk of overgeneralizing findings from a single system.

Second, the surveyed participants were predominantly healthcare professionals affiliated with specific institutions, which may introduce bias and limit the applicability of the findings to broader or more diverse populations. Third, ChatGPT’s reliance on publicly available data, with a cutoff in September 2021, constrains its ability to address advancements in rapidly evolving fields like SCS. This limitation underscores the need for continuous updates to training datasets to ensure relevance in clinical practice.

Finally, this study did not assess patient perceptions directly, instead relying on evaluations by healthcare professionals and non-clinical participants. Future research should include patient-focused assessments to better understand the practical utility of AI-generated responses in real-world clinical settings.

### 4.2. Perspectives

Looking ahead, integrating AI tools like ChatGPT into standard clinical practice for SCS and other interventional pain procedures holds significant promise. By automatically generating clear and accessible patient education materials, AI can reduce clinicians’ workload, streamline communication, and enhance overall patient satisfaction. However, to realize these benefits, developers and healthcare institutions must focus on key issues. For example, it is mandatory to prioritize frequent updates of AI training data to reflect the latest clinical guidelines and technologies. Additionally, it requires the adaptation of AI outputs to cultural, linguistic, and individual patient needs as well as collaboration between AI developers, regulatory bodies, and healthcare professionals to establish robust frameworks for accountability and quality control [44].

Ultimately, the future of patient education in chronic pain management may hinge on AI’s ability to offer timely, evidence-based, and person-centered resources that are seamlessly integrated with physician-led care [45]. Nevertheless, as Stengel et al. [46] suggested, an LLM can not only be used to answer questions but also to formulate them. As continuous refinements are made, AI-powered systems have the potential to become indispensable allies in delivering patient-centered neuromodulation services and improving clinical outcomes.

## 5. Conclusions

This study demonstrates that ChatGPT has significant potential as a supplementary tool for patient education in SCS. Due to its reliable, accurate, and comprehensible responses to general queries highlighted, the chatbot can be implemented to simplify complex medical information and support patient understanding. However, the AI’s limitations in addressing highly technical or context-specific topics and its reliance on an outdated knowledge base underscore the need for further refinement and validation.

To maximize its clinical utility, AI tools like ChatGPT should be regularly updated with domain-specific and regionally relevant data to ensure their accuracy and relevance in rapidly evolving fields like SCS. Additionally, integrating AI into clinical workflows must prioritize its role as an adjunct to healthcare professionals, who are essential for tailoring information to individual patient needs and navigating complex clinical scenarios.

Future efforts should enhance AI capabilities through domain-specific training, expand datasets to reflect current knowledge, and validate their effectiveness in diverse patient populations. By complementing, rather than replacing, physician-patient interactions, AI-based tools have the potential to improve patient engagement, adherence, and outcomes in advanced therapies like SCS.

## Figures and Tables

**Table 1:** Common patient questions about spinal cord stimulation (SCS) categorized by procedural domain.

Domain	Questions
Pre-Implant	Q1. What is spinal cord stimulation?
	Q2. How does SCS work?
	Q3. Is spinal cord stimulation (SCS) painful?
	Q4. What are the risks and benefits?
Implant	Q5. What can I expect during and after the procedure?
	Q6. How is the SCS device programmed?
	Q7. What does the stimulation feel like?
	Q8. What are the different types of waveforms used in SCS?
Post-Implant	Q9. How long does the battery last, and what happens when it needs to be replaced?
	Q10. Can the SCS device be turned off or removed if necessary?
	Q11. What activities should I avoid with an SCS device?
	Q12. How soon can I return to normal activities after the procedure?
	Q13. What should I do if I experience discomfort or complications with my SCS device?

**Table 2:** Reliability, accuracy, comprehensibility, and word length of ChatGPT responses to SCS questions.

Question	Domain	Reliability (Mean ± SD)	Accuracy (Mean ± SD)	Comprehensibility (Mean ± SD)	Word Count (Mean ± SD)
Q1	Pre-procedural	5.2 ± 0.6	2.8 ± 0.2	2.9 ± 0.1	155 ± 18
Q2	Pre-procedural	5.0 ± 0.8	2.7 ± 0.3	2.7 ± 0.3	145 ± 22
Q3	Intra-procedural	5.6 ± 0.5	2.8 ± 0.1	2.9 ± 0.1	185 ± 20
Q4	Intra-procedural	5.5 ± 0.6	2.7 ± 0.2	3.0 ± 0.0	190 ± 25
Q5	Post-procedural	5.0 ± 0.7	2.9 ± 0.1	2.8 ± 0.2	210 ± 28
Q6	Post-procedural	5.3 ± 0.6	2.7 ± 0.2	2.9 ± 0.1	205 ± 25
Q7	Post-procedural	4.6 ± 0.8	2.6 ± 0.3	2.7 ± 0.2	195 ± 22
Q8	Post-procedural	5.1 ± 0.7	2.8 ± 0.2	2.9 ± 0.1	200 ± 24
Q9	Post-procedural	5.5 ± 0.5	2.7 ± 0.2	3.0 ± 0.0	180 ± 20
Q10	Post-procedural	5.2 ± 0.6	2.5 ± 0.3	2.6 ± 0.4	210 ± 30
Q11	Post-procedural	5.4 ± 0.5	2.9 ± 0.1	2.8 ± 0.2	195 ± 23
Q12	Post-procedural	4.8 ± 0.9	2.6 ± 0.2	2.7 ± 0.2	185 ± 25
Q13	Post-procedural	4.5 ± 0.7	2.6 ± 0.4	2.5 ± 0.4	220 ± 35

Notes: Reliability scores range from 1 (lowest) to 6 (highest), accuracy scores from 1 (lowest) to 3 (highest), and comprehensibility scores from 1 (lowest) to 3 (highest). Word count represents the average number of words per response with standard deviations.

## Data Availability

Data collected and generated for this investigation are available from the first author (G.L.).

## Abbreviations

The following abbreviations are used in this manuscript:

SCS	Spinal cord stimulation
AI	Artificial Intelligence
GPT	generative pre-trained transformer
LLM	Large language model
ICU	Intensive Care Unit
